# Surgery

**Published:** 1894-05

**Authors:** 


### SURGERY.

EDITED BY DR. F. W. McRAE.

### TREATMENT OF GRANULATING WOUNDS.

Van Arsdale (*New York Medical Journal*) holds that granulating wounds should be treated much in the same manner as are primary aseptic ones. Iodoform gauze, and over this a layer of cotton and a bandage, constitutes the dressing commonly applied to granulating wounds. As a consequence of this dressing inflammation is increased from the accumulation of secretion on the wound and on the surrounding tissue, conditions for which the familiar word retention may be used. A further disadvantage of the iodoform gauze is found in the fact that it sticks to the wound and cannot be removed without lacerating the granulations.

The form of retention just described may be obviated by dampening the dressing with some aqueous solution and covering it with protective impermeable to air. This dressing is in general use for some infected and sloughing wounds, where it certainly renders excellent service. For general use the moist dressing is not satisfactory, since from its warmth and moisture it acts as a poultice, increases the secretion from the wound, causes the granulations to become exuberant, brings about eczematous conditions of the skin, etc. Acute œdema with exfoliation of the epidermis is of common occurrence. The proliferation of all kinds of bacteria in moist dressings soon renders them putrid, hence they must be frequently changed.

Certain wounds may be treated in a manner somewhat similar to that employed in securing healing under Schede's moist blood-clot. A piece of gutta-percha tissue is placed over the wound; over this absorbent material is held in place by a bandage. Wounds treated in this manner, as seen by the author, have not run a satisfactory course.

Oil dressings are objectionable for obvious reasons. Castor oil is, however, an exception to the rule; it is soluble in alcohol, it will take up fifty per cent. of Peru balsam, it is viscid enough to remain for a long time in contact with the wound, and will remain in those portions of the dressing on which it was originally spread. It does not prevent absorbent gauze from taking up the secretions of the wound, and thus does not interfere with drainage. However much a granulating wound may discharge, the wound always appears clean and dry when the oil dressing is removed. The oil does not appear to turn rancid when mixed with Peru balsam.

To sterilize oil it is necessary to subject it to a temperature of 160° C. for two hours. For general use a four or five per cent.

solution of balsam in the oil is sufficient. Its application to a wound is simple. A bunch of plain sterilized gauze is spread with this solution over an area somewhat larger than the wound to be dressed; this is most readily accomplished by means of a large brush. The solution should permeate from four to six layers of the gauze. The gauze is now simply laid on the wound, so that the oil comes in contact with it; then a protective layer of rubber tissue or oil paper is spread over all and then the bandage applied. Any of the antiseptic or astringent powders can first be dusted over the wound; the oil dressing will then prevent the formation of a crust. As a dusting powder subiodide of bismuth is to be preferred. The following combination is frequently used:

| | | |
|---|---|---|
| ℞ | Balsam of Peru | gr. xx. |
| | Iodoform | gr. x. |
| | Castor oil | f℥j. |

This dressing need not be changed oftener than twice a week. It does not actively prevent suppuration; it simply drains the wounds and keeps them in a clean condition.—*College and Clinical Record.*

---

### THE PRESENT STATUS OF THORACIC SURGERY.

Gaston (*Jour. of the Amer. Med. Assn.*), after discussing the various methods proposed, draws the following inferences:

1. All penetrating wounds of the thorax may be closed hermetically by suture or otherwise, after allowing the discharges of fluid blood from the opening.

2. Foreign bodies lodged in the bronchi may be removed by incision of the trachea at the lowest available point.

3. Experiments on reaching the bronchi through the chest wall afford little encouragement in undertaking operations upon the human subject.

4. Medication as a preventive and a curative agency in pleuritic effusion is worthy of trial before having recourse to aspiration.

5. Aspiration is indicated when there are large serous accumulations in the chest, and likewise in pneumo-thorax, but cannot be relied upon for the relief of purulent collections.

6. Partial resections of ribs are attended with better results in some cases of empyema than the complete removal of the segments of several ribs.

7. The excision of a small portion of one rib with the introduction of drainage-tube has been generally attended with good results.

8. Washing out the cavity of the chest is not requisite, except in contamination and decomposition of the contents.

9. The operation of thoracotomy for abscess and gangrene of the lung should be accompanied with antiseptic applications and with tamponage of gauze.

10. Tumors of the mediastinum may admit of interference, but further developments of technique are necessary before the method can be generally advised.—*Therapeutic Gazette.*

---

### Total Extirpation of the Prostate Gland.

Dittel, of Vienna, reported a case of total extirpation of the prostate, before the Vienna Medical Association.

This operation has only been performed three times with *un*successful result.

Dittel performed the operation successfully on a man thirty-two years of age. The patient came under his observation suffering with hematuria and difficulty of micturition.

The diagnosis of stone in the bladder was excluded.

An oval swelling could be felt through the abdominal wall, even after evacuation of the bladder, and through the rectum the very much enlarged gland could be felt. With the endoscope a bleeding prominence could be noticed on the right wall of the bladder.

*Operation: Sectio Alta*—Removal of the prominence with the sharp scoop. Incision over the coccyx to the anus. Extirpation of coccyx. The prostate reached to the right of the rectum, the capsule opened and the swollen gland loosened from the capsule. Upon filling the bladder with liquid, none of it was evacuated into the cavity of the wound. The urethra was not injured at all.

Microscopically the swelling appeared to be a fibroma, but could not be exactly classified microscopically.

The excellent result was due to the favorable anatomical relations. The urethra in this case did not pass through the gland but before it.

Dittel observed the same relation on three cases.

*Conclusions:* 1. In all cases, where the swelling of the prostate surrounds the urethra, extirpation is impossible without injury to the urethra. Possibly the swelling can be divided and each half loosened, if practicable, from the urethra.

2. In every case the sacral method promised better results than the perineal.—*Internat. Clin. Rundschau. Translated by Dr. L. Amster, Atlanta.*

---

### THE PRESENT POSITION OF GALL-BLADDER SURGERY.

Czerny, after a general consideration of gall-bladder surgery, presents the following conclusions:

1. Gall-stones require operation, if they cause frequently repeated or lasting trouble.

2. Empyema of the gall-bladder imperatively demands operation, as does hydrops, if it gives serious trouble.

3. The typical operation for gall-stones consists in incision, removal of the stones, and suture of the gall-bladder; in this, however, the abdominal wound should be drained for a short time.

4. If the cystic duct is closed, if the gall-bladder is the seat of inflammation, or the contents are greatly altered, then a temporary gall-bladder fistula must be made.

5. Extirpation of the gall-bladder is indicated only in cases of severe inflammatory carcinomatous involvement.

6. When the ductus choledochus is closed, the operation is absolutely indicated, if the strength of the patient will permit. If one does not succeed in removing the stone or obstruction, then it is recommended to produce a fistula between the gall-bladder and duodenum.

7. The best incision for gall-bladder operations is an ⌋ shaped cut, the vertical limb lies in the linea alba, and the horizontal part runs towards the right, just below the level of the umbilicus.

8. The danger to life of gall-stone operations will be probably less than in operations for vesical calculus.—*Epitome of Medicine.* —*Buffalo Medical and Surgical Journal.*

